# Useful Recipes

**Published:** 1861-07

**Authors:** John R. Cushing

**Affiliations:** South Butler, Alabama


### ARTICLE II.

*Useful Recipes.* Selected by John R. Cushing, M. D., South Butler, Alabama.

Mr. Editor:

With this I send some valuable Recipes, many of which I have used in my own practice. I take them from my common-place book, and at random, and without classification. Some of them may be useful to the many readers of the *Journal*, and having them published will be a ready reference in case of *hurry*, and a change of prescription:

Tinctures.—*Tinct. Arnica.*

℞. Arnica Flowers, ℥ iv. }

" Alcohol, . . . . . . . . O. i. } Digest together express and filter.

---

Saturated Tinct. Sangunaria.

℞. Pulv Sangunaria, ℥ iv. }

" Alcohol, . . . . . . . . O. i. } Dose 30 to 40 gtts.

---

Sat. Tinct. Blk. Snakeroot.

℞. Blk. Snake-Root, ℥ iv. }

" Alcohol. . . . . . . . . O. i. } Dose, 30 to 40 gtts.

### Tinct. Phosphorus.

℞. Phosphorus, (Pure,) grs. x.
" Anhydrous Alcohol, ℥ ij.

Put in vial and disolve by means of a water-bath—keep well stopped in colored vial.—*Dr. Ames rec.*

---

### Liniment.

| | |
|---|---|
| ℞. Tr. Arnica, ℥ ij.<br>" Belladon, ℥ ij.<br>" Digitalis, ℥ ij. | An excellent liniment for nervous pains, neuralgia etc. *Dr. Fowler, Ala.* |

---

| | |
|---|---|
| ℞. Vol. Liniment, ℥ iv.<br>" Chloroform,.... ℥ i.<br>" Tr. Aconite,.... ℥ i. | For neuralgia, sciatica, etc., *Dr. Fowler.* |

---

| | |
|---|---|
| ℞. Chloroform, ʒ iv.<br>" Tr. Digitalis, ʒ viii.<br>" " Belladonna, ʒ viii.<br>" " Comphora, ʒ viii.<br>" " Catnharidis, ʒ iv.<br>" " Olive,..... ʒ viii | One of the best liniments, for all nervous, rheumatic and neuralgic pains—a favorite liniment of mine. |

---

### Miscellaneous Recipes.

| | |
|---|---|
| ℞. Vin. Ergota,......... ℥ ss.<br>Tr. Buchu,.......... ℥ i.<br>Vin. Colchi,......... ℥ ss.<br>Bal. Copab,......... ℥ ij.<br>Pulv. Acacia,....... ℥ ss.<br>Syr. Simplex,...... ℥ iij.<br>Ol. Wintergreen.....gtt xxv. | For Gonorrhœa and other urinary diseases. The Ol. Wintergreen covers the nausious taste of the Copavia. Dose, teaspoonful three times a day. |

---

℞ Mur. Ammonia,.........grs lxiv.

Tr. Seneka,............. ʒ viii.

" Lobelia,............. ℥ ss.

Sy. Yellow Dock, ft...... ℥ iv.

Tea-spoonful every three hours as an Expectorant.

℞ Iod. Potassium..... ℥ i.

Donovans Solution. ʒ vi.

Tr. Strychnine..... ʒ vi.

Ext. Sarsap........ ℥ i.

Syr. Simplex....... ℥ vi.

Ext. Taraxicum..... ℥ ss.

Dilut. Alcohol...... ℥ ij.

Aqua. Pura ft...... ℥ xx.

For long standing chills and fever—Dose, tea-spoonful three times daily, after eating.

---

℞ Poke Root....¼ lb.

Burdock......¾ "

Sarsaparilla...¼ "

May-Apple....2 ozs.

Blood-Root....1 oz.

Best Whisky..1 qt.

This is Dr. Ames' celebrated receipe for the cure of scrofulous diseases; it has been tried by many, and has met with great success. The Dose is a tea-spoonful, before eating, and never increase the dose.

---

℞ Tr. Aloes, Comp'd.. ℥ ij.

Tr. Gentian........ ℥ i.

Tr. Rhei........... ℥ i.

Ext. Colocynth.... ʒ i.

Ext. Taraxicum.... ʒ ij.

Bi Chlo. Hydrag..grs. iij.

Syr. Sarsap........ ℥ iij.

Aq. Pura ft....... ℥ x.

Mix thoroughly in a mortar.

An excellent alterative for costiveness, especially when complicated with biliary derangements. Dose, tea-spoonful three times a day, before or after eating, one hour. J. R. C.

---

℞ Fowler's Sol.....gtt. xlviii.

Tr. Aconite.....gtt. xxxii.

Ag. Destilat..... ℥ ij.

For dry Bronchitis; the dose for an adult is, one-third tea-spoonful every three hours.
Dr. Fowler.

---

℞ Sat. Tinct. Sangumaria, ℥ i.

Tinct. Opii............ ʒ ij.

Vin. Ipicac vel. Antim. ʒ vi.

Sy. Tolu,............ ℥ ij.

Mix.

Dose 30 to 40 gtts., an admirable expectorant in Cattarrhal affections, and in casesof troublesome cough, following influenza, it is given with much advantage.

*J. R. C.*

| | | |
|---|---|---|
| ℞ Pulv. Sanguinaria,<br>" Rhei, . . . . . .<br>" Castile Soap, | aa. ʒ i.<br>℈ ij. | Make into 32 pills. Dose, one, night and morning. A capital remedy in habitual costiveness. |

---

| | | |
|---|---|---|
| ℞ Sat. Tinct. Bl'k Snakeroot, | ℥ i. | For Rheumatism Anasarca, and Dropsies generally. A Tea-spoonful thrice daily. |
| Iod. Potass. . . . . . . . . . . . . . | ʒ ij. | |
| Syr. Ipicac, . . . . . . . . . . . . . | ℥ i. | |
| Aq. Pura., . . . . . . . . . . . . . . | ℥ ij. | |

---

| | | |
|---|---|---|
| ℞ Ergota Pulv., . . . . . . . . . | ʒ i. | For uterine hemorrhage. Dose, *pro re nata*. *Parish's Forumlary*. |
| Alum or Borate Soda. | ℈ j. | |
| Mix and divide in powders. No. vi. | | |

---

℞ Ext. Colchi Acetici ʒ ss.; Pulv. Doveri, ʒ iss. and vi. grs. Divide into xxiv pills.—Dose, two at night and one before breakfast and dinner. For Rheumatism.—*Ibid.*

---

| | |
|---|---|
| ℞ Sulp. Quinia, . . . . . . . . . . . . . . . . . . . . . . . | xlv grs. |
| Pulv. Digitalis, . . . . . . . . . . . . . . . . . . . . . . | xv grs. |

Make xxx pills. Dose, one night at bed time. A good remedy for nervous or sick headache.

---

| | | | |
|---|---|---|---|
| ℞ Tannic Acid, | grs., | xx | Excellent for Diarrhœa and mucous Dysentery in children. Dose, gtt. xx every hour or two until symptoms are relieved. |
| Spts. Terebinth, | gtt. | xlviii | |
| Sulph. Acid, . . | gtt. | xii | |
| Tinct. Opii, . . . | gtt. | xxx | |
| Pulv. Acacia, . | ʒ | ij | |
| Syr. Simplx. ft. | ℥ | ij | |

---

| | | |
|---|---|---|
| ℞ Tr. Strychnine, | ʒ i | For Gonorrhœa and Gleet—good. *J. R. C.* |
| Vin Ergota, . . | ʒ ss | |
| Mur Ammon., | ʒ ij | |
| Aqua. Cin'm'n., | ℥ ij | |

Dose—Tea-spoonful thrice daily.

---

| | | | |
|---|---|---|---|
| ℞ Sulph. Magnes., | grs. | xxx | For Diarrhœa in children. Dose, teaspoonful after every operation. |
| Sulp. Acid, . . . . | gtt. | iv | |
| Tr. Assafœt., . . | gtt. | xxx | |
| Tr. Opii, . . . . . . | gtt. | iv to viii | |
| Aq. Cinnam. ft. | ℥ | ij | |

| | |
|---|---|
| ℞ Tinct. Belladon.,. ʒ ij<br>Tinct. Aconite,... ʒ ij<br>Tinct. Strychnine ʒ iij | For painful menstruation. Dose, six drops four times a day, gradually increased up to eight drops, to be taken in a little water. |

---

| | |
|---|---|
| ℞ 2 parts Collodion<br>1 part Castor Oil. | For sore nipples. It produces an artificial cuticle. Also excellent for burns. |

---

| | |
|---|---|
| ℞ Vin. Cholchicum,.. ℥ i<br>Vin. Ergota,...... ℥ i<br>Am. Tinct. Valerian, ℥ ij<br>Tinct. Sanguinaria, ℥ i<br>Tr. Aconite,......gtt. lx<br>Mix. | For uterine Engorgement, Dysmenorrhœa, and Lencorrhœa. Dose, tea-spoonful thrice daily. Its use should not be suspended, except at intervals, for many weeks, until a cure is made. A capital remedy. *J. R. C.* |

---

| | |
|---|---|
| ℞ Chlorate Potass, vi grs.<br>Aqua Pura,...... ℥ i. | Injection for Gonorrhœa. |

---

**Judkin's Ointment.**

℞ Linseed oil one pound, heat until it turns a feather black; then add four onces of Red Lead; gradually stir till all is mixed well; then add gradually Spirits Turpentine half pound, and one ounce Sugar Lead—stir all together until cold. One of the best unguents for old sores, weak joints, etc.

---

**Creosote.**

℞ Creosote, gtt........... v.
Aqua Pura,........... ℥ i

For Tetter, Chilblains, Erysipelas, ill-conditioned ulcers of the surface and throat; also in profuse ptyalism, applied with a camel-hair pencil. The internal use is from 1 gtt. up to xx. As an anti-emetic, dose, ½ to 2 gtt. in a table spoonful of water. This is excellent in bloody vomiting. Creosote is very much neglected.—*Mitchell's Therapeutics.*

**Milk of Assafoetida.**

Take a lump of the Gum about the size of a nutmeg; put it into a cup of hot water; then add a tea-spoonful of Pulv. Gum Acacia; then put into a well corked bottle—Sugar may be added. Dose for an adult, table-spoonful, freqently repeated. It is a capital article for drunkards who wish to quit the intoxicating fluid. It calms nervous irritation of the whole system, and, at length, acting gently on the bowels, no fear need be entertained of giving too much; and, according to Prof. Mitchell, it is better for young children than any of the opiates. The worst of infantile colics is speedily relieved by doses of the milk, by the mouth, and injection. In absence of the milk, the tinct. will do. I have discarded opiates altogether, *nearly*, in diseases of children; for I beleive the indiscriminate use of those drugs has sent thousands of children to the grave. Also this preparation is much neglected.

---

**Rose Pills, or, Anti-Dyspeptic Pills.**

| | | |
|---|---|---|
| Day Pills. | | Dose, one, three times a day, before eating. The pills must be made into mass with the Oil-of-Cloves. The pills ordinary size. |
| ℞ Bi. Carb. Soda, | Equal parts. | |
| Aloes Pulv.... | | |
| Castile Soap... | | |

---

| | |
|---|---|
| Night Pills. | |
| ℞ Mass Hydrarg | Equal parts. |
| Aloes Pulv... | |

To be made in three-grain pills. Does, one every night at bed time. The practitioner will find this old and excellent recipe, good in all derangements of the hepatic and digestive functions. This is a favorite preparation of Prof. Knott, of Griffin, Georgia, from whom I got the recipe, and I can indorse all his predilections in regard to it.

---

| | | |
|---|---|---|
| ℞ Acid Hydrocyanic | ʒ ij | Applied for Pruritus and Erysipelas, several times a day, as well as in many other herpetic diseases. |
| Act. Plumbi., grs. | xvi | |
| Alcohol,......... | ℥ ss | |
| Aqua,......... | ℥ viii | |

*Mitch. Ther. pp.* 58.

**Ring-Worm and Tetter Lotion.**

| | | |
|---|---|---|
| ℞ | Tart. Emetic, ..... | xl grains. |
| | Corosive Sublimate | |
| | Acitate Plumbi,... | |
| | Tinct. Opii, gtt. xl. | |

Mix well in two ounces of Aqua Pura, and apply to Ringworm two or three times a-day until it produces an increase of soreness of the part.—*Fort.*

This has only to be used and it will become a favorite.

---

**For Dysentery.**

℞ Sulp. Magnesea, ...... ʒ i

Dilut. Sulph. Acid, gtt. xx

Every four hours, or pro re nata, if there be tenesmus at night, and severe, give pill Hyd. grs. iv, Pulv. Opii gr. i. Allow only lightest kind of diet; no stimulants of any kind. Patient to be kept in the recumbent position, with (in severe cases,) the thighs elevated with a pillow, to relax abdominal muscles, with a mustard poultice every twelve hours over the abdomen. The sagacious practitioner will readily discover the *rationale* of this treatment. I have used it with great success; it originated with Dr. Ford, of London, and used by him successfully in India.—*See Rankin's Abt. No.* 30, *p.* 95, *Am. Ed.*

---

**Treatment of Dysmenorrhœa.**

2 parts Ver. Veride; 6 parts Tinct. Cantharides, made of double strength. Dose—Begin with 8 drops, increasing 2 drops every week up to 16 drops.

---

**Sarsaparilla Syrup.**

| | | | |
|---|---|---|---|
| ℞ | Compound Sarsaparilla Syrup, | oi | A good Alterative. |
| | Bichlor. Hydrog.,............ | grs. ij | |
| | Ext. Conium.,............... | ʒ i | |

---

**For Nervous Headache.**

℞ Sulph. Quinia,................ grs. xlv
Pulv. Digitalis,................ grs. xv

Make into xxx pills. Take one every night, a tbed time.

### For Gonorrhœa.

In cases after inflammatory symptoms have been reduced, and the disease has become chronic, or a gleet, the following recipe will be found good.

| | | |
|---|---|---|
| ℞ | Creosote, gtt. .................... | x |
| | Acid Tanic, grs.................... | x |
| | Aqua.,.......................... | ℥ iv |

Inject four times a day, retaining each injection a minute or two, first washing out the urethra with an injection of cold water, before using it.

The following Balsam mixture may be given:

| | | | |
|---|---|---|---|
| ℞ | B. Copavia,...... | aa. ℥ i | Tea-spoonful morning and noon, and two at bed time.—*N. O. Jour.*, *Sept.*, 1858, *have used it and indorse.* |
| | Spts. Lavander Co. | | |
| | Aqua. Calcis,..... | | |
| | Tinct. Opii, ........ | ʒ ij | |
| | Sach. Alba.,........ | ʒ iv | |

